# New Publications Received

**Published:** 1859-09

**Authors:** 


*New Publications Received.*

Braithwaite's Retrospect of Practical Medicine and Surgery. Part the Thirty-ninth. W. S. Townsend & Co., 46 Walker Street. This admirable work is unusually abundant in interesting matter.

An Essay on Intermittent and Bilious Remittent Fevers: with the Pathological Relation to Ozone. By E. S. Gaillard, M. D. From the Author.

A Memoir on the Treatment of Epidemic Cholera, read before the members of the French Academy of Sciences, with their Report thereon. By Joseph Ayre, M. D., Member of the Royal College of Physicians of London and Edinburgh, and many years Physician to the General Hospital at Hull. London: J. Churchill, New Burlington Street.

The British and Foreign Medico-Chirurgical Review; a Quarterly Journal of Practical Medicine and Surgery. July, 1859. Republished by S. S. & W. Wood, 389 Broadway.

We have also received two new medical exchanges—"*The Dental Cosmos*" and the "*Cleveland Medical Gazette.*" The former is a new series of the "*Dental News Letter,*" and presents an elegant appearance. The latter is a new monthly, of thirty-two pages, edited by Gustav C. E. Weber, M. D., Professor of Surgery in the Cleveland Medical College. We welcome them to our exchange list.

*Five Essays.* By John Kearsley Mitchell, M. D., late Professor of Practice of Medicine, in Jefferson Medical College of Philadelphia; Member of the Academy of Natural Sciences, of Philadelphia; Fellow of the Philadelphia College of Physicians, etc. Edited by S. Weir Mitchell, M. D. Lecturer on Physiology in the Philadelphia Association for Clinical Instruction. Philadelphia: J. B. Lippincott & Co., 1859.

The little volume which is now before us is a pleasant remembrancer of its distinguished author, whose death we have so lately chronicled. Dr. Mitchell was truly a great man; and the immense number of the profession who have listened to his teaching, and all of whom must have carried away with the precepts inculcated in the lecture-room grateful and affectionate recollections of the teacher, will welcome this volume as a souvenir of their former friend. Those who have not been brought into contact with the author, will find in these essays strong and original ideas, elegantly presented, and cannot fail to be both interested and instructed by their perusal.

The first essay is on the "Cryptogamous Origin of Malarious and Epidemic Fevers," with which the profession is familiar. Then follow essays on "Animal Magnetism, or Vital Induction," "On the Penetrativeness of Fluids," "On the Penetrativeness of Gases," and "On a New Practice in Acute and Chronic Rheumatism." All of these essays have appeared before, with the exception of that upon animal magnetism. They have been edited by Dr. S. Weir Mitchell, who has dedicated them to the graduates of the institution with which his father was so long connected.

